# Evaluation of Hearing Thresholds in Infants With Autism Spectrum Disorder Using Auditory Brainstem and Steady-State Responses

**DOI:** 10.7759/cureus.77537

**Published:** 2025-01-16

**Authors:** Dimitra Chaldi, Konstantinos Mourtzouchos, Spyridon Lygeros, Gerasimos Danielides, Stefanos Naxakis

**Affiliations:** 1 Department of Otolaryngology - Head and Neck Surgery, School of Medicine, University of Patras, Patras, GRC; 2 Department of Otorhinolaryngology, Karamandaneio Children's Hospital of Patras, Patras, GRC

**Keywords:** auditory brainstem response, auditory steady-state response, autism spectrum disorder, pediatric hearing evaluation, speech delay

## Abstract

Introduction

In recent years, most studies on the hearing abilities of children with autism spectrum disorder (ASD) have used the auditory brainstem response (ABR) test alongside other audiological assessments, while only a few have explored the auditory steady-state response (ASSR) test. Moreover, these two electrophysiological methods have not yet been directly compared in this population. This study aims to compare click ABR and chirp ASSR in children with ASD and speech delays to determine whether differences in hearing thresholds can be identified.

Methods

Children with ASD and speech delays referred to Karamandaneio Children’s Hospital between December 13, 2019, and December 6, 2023, were retrospectively identified as cases. Children diagnosed with speech delays but without ASD, who were referred to the same hospital during the same period, were included as controls.

Results

This study evaluated 30 children (21 males and nine females, totaling 60 ears). Of these, 20 children had been diagnosed with both ASD and speech delay, while 10 were non-ASD children with speech delay and normal hearing, serving as the control group. The participants were aged 19-68 months (median age = 38.5). All ears that responded to the click ABR also responded to each chirp frequency tested by the ASSR. This finding highlights the additional information provided by the ASSR compared to the ABR for threshold estimation, as no instances were observed where a response was obtained for the ABR stimulus but not the ASSR. Click ABR thresholds showed a statistically significant association with chirp ASSR threshold averages at 1,000, 2,000, and 4,000 Hz (rho = 0.316, p = 0.014), as well as at 2,000 and 4,000 Hz (rho = 0.277, p = 0.032). The strongest positive correlation was observed between the two chirp ASSR threshold averages (rho = 0.971, p < 0.001).

Conclusions

The results suggest that participants diagnosed with ASD exhibit statistically significantly lower mean scores for both click ABR and chirp ASSR threshold averages, with the effect being slightly more pronounced for the chirp ASSR thresholds than for the click ABR.

## Introduction

Autism spectrum disorder (ASD)

ASD is associated with a combination of genetic and environmental factors and affects how the brain processes information, as well as the connection and organization of synapses with nerve cells. The underlying mechanisms are complex and not fully understood [1]. Autism is not classified as a psychiatric disorder but falls under the category of pervasive developmental disorders, which are characterized by deficits in various areas of an individual’s development, hence the term “pervasive” [2]. The term “autism” is derived from the Greek word “autos,” meaning “self,” and, according to Paul Eugen Bleuler, refers to the morbid self-admiration and withdrawal of the individual into themselves [3].

According to the American Psychological Association, autism is a neurodevelopmental disorder marked by impairments in verbal or nonverbal communication and diminished social interactions. Additionally, individuals with autism often have restricted interests and engage in repetitive or stereotypical behaviors. They may also display reduced empathy, difficulty with imitation, lack of social play, language delays or absence of verbal communication, and a preference for maintaining stability [4]. These features typically emerge before the age of three, although they vary significantly between individuals depending on factors such as developmental level, language abilities, and chronological age.

Autism and speech delay

Children are expected to reach certain developmental milestones as they grow, such as forming simple two-word sentences by the age of two or experiencing a vocabulary explosion by 18 months. A failure to reach these milestones, or to develop skills appropriate for the child’s age, may signal a developmental disorder, including autism. Some individuals with ASD may begin to communicate at a later-than-typical age or may remain nonverbal throughout their lives. Others may acquire minimal communication skills, learning to produce specific words and sentences, but may struggle to use them effectively.

Delayed speech is common not only in children with autism but also in those with typical development. Thus, while any language delay is a cause for concern, it is not necessarily an indication of autism [5]. However, speech delay in children with autism differs significantly from other delays, as it typically coexists with other communication challenges, such as not using gestures, failing to respond to their name, and a lack of interest in socializing with others.

Although ASD is usually not diagnosed until the age of two to four years, children may exhibit signs of the communication difficulties characteristic of autism within the first year of life, such as atypical development or behaviors that suggest an early childhood autism diagnosis [6,7]. Additionally, children between 12 and 24 months often display a “reduced” percentage of attentive communication skills and may struggle to engage with others about their interests. Furthermore, children with ASD tend to have a lower rate of babbling and other preverbal sounds, including phonemes and atypical unintelligible vocalizations. Generally, children with ASD begin to acquire expressive language or use words as their primary form of communication between the ages of two and six years. The age at which a child first uses words can predict later cognitive and adaptive skills [8].

Autism and hearing

Although studies suggest a link between hearing loss and autism, the precise relationship between the two remains unclear. While some studies report a higher prevalence of hearing difficulties in individuals with ASD, others find no such association [9]. However, it has been established that approximately 50-86.7% of individuals with ASD experience auditory processing difficulties [10]. As such, the auditory system is of particular interest when examining sensory processing in autism.

Research indicates that some children with autism perform worse on tests of auditory function, such as auditory temporal processing, spatial listening, and functional hearing, compared to typically developing children, despite having a normal pure-tone audiogram [11]. Specifically, studies have shown that these children may experience either under- or over-sensitivity to sound stimuli, difficulty processing spoken commands, and impaired speech recognition in noisy environments [12]. In contrast, research by Rosenhall et al. [13] suggests that severe hearing loss in children with ASD is rare. Nevertheless, the precise link between autism and hearing loss remains uncertain.

The effects of hearing loss are well documented, and when combined with an ASD diagnosis, hearing loss becomes another potential co-morbid condition. Hearing difficulties can exacerbate the features of ASD. Children with ASD are often diagnosed with hearing loss, so it is crucial to consider how autism and hearing loss may interact. Children with ASD often experience hearing difficulties at frequencies around 2,000 Hz, which can result in distorted speech perception [10].

Hearing evaluation in autism

Delayed speech development and hearing disorders are two potential early indicators of autism. Parents of children with ASD and speech delay often suspect hearing loss as the cause, making a hearing evaluation an essential first step toward diagnosing autism. According to the CDC, diagnosing potential hearing loss in a child involves two steps: an early audiological screening and a comprehensive audiological assessment [14]. For typically developing infants, screening typically includes objective tests such as the otoacoustic emission (OAE) test and the automated auditory brainstem response (ABR) test. These objective tests may be supplemented with subjective tests, such as behavioral observation audiometry, conditioned play audiometry, and visual reinforcement audiometry, although results can be unreliable in children with reduced attention span or behavioral issues. Once children reach age four, standard pure-tone audiometry is generally used to assess hearing in typically developing children.

However, these audiological tests are often difficult to administer successfully in children with developmental disabilities or other challenges. In infants (up to around seven to eight months of age), reliable behavioral test results are also hard to obtain. To address this challenge, various tests, such as the ABR and the auditory steady-state response (ASSR), can be used in children who are unable or unwilling to complete subjective hearing tests. The clinical comparison and evaluation of these two electrophysiological methods are of particular interest for both research and clinical applications, as they may serve as valuable diagnostic tools in the early identification of autism in children.

In this study, we assess the reliability and validity of ABR and ASSR in this specific clinical population. While several studies have explored ABR and its role in autism, no study has yet compared the accuracy of both methods in this population. Therefore, the primary aim of this study was to examine and compare the findings from click ABR and chirp ASSR in a relatively large sample of children diagnosed with ASD and speech delay. The results were analyzed to assess the usefulness of these methods, the potential risk of central auditory processing issues, and the implications for initiating early intervention.

## Materials and methods

Subjects and methods

**Figure 1:**
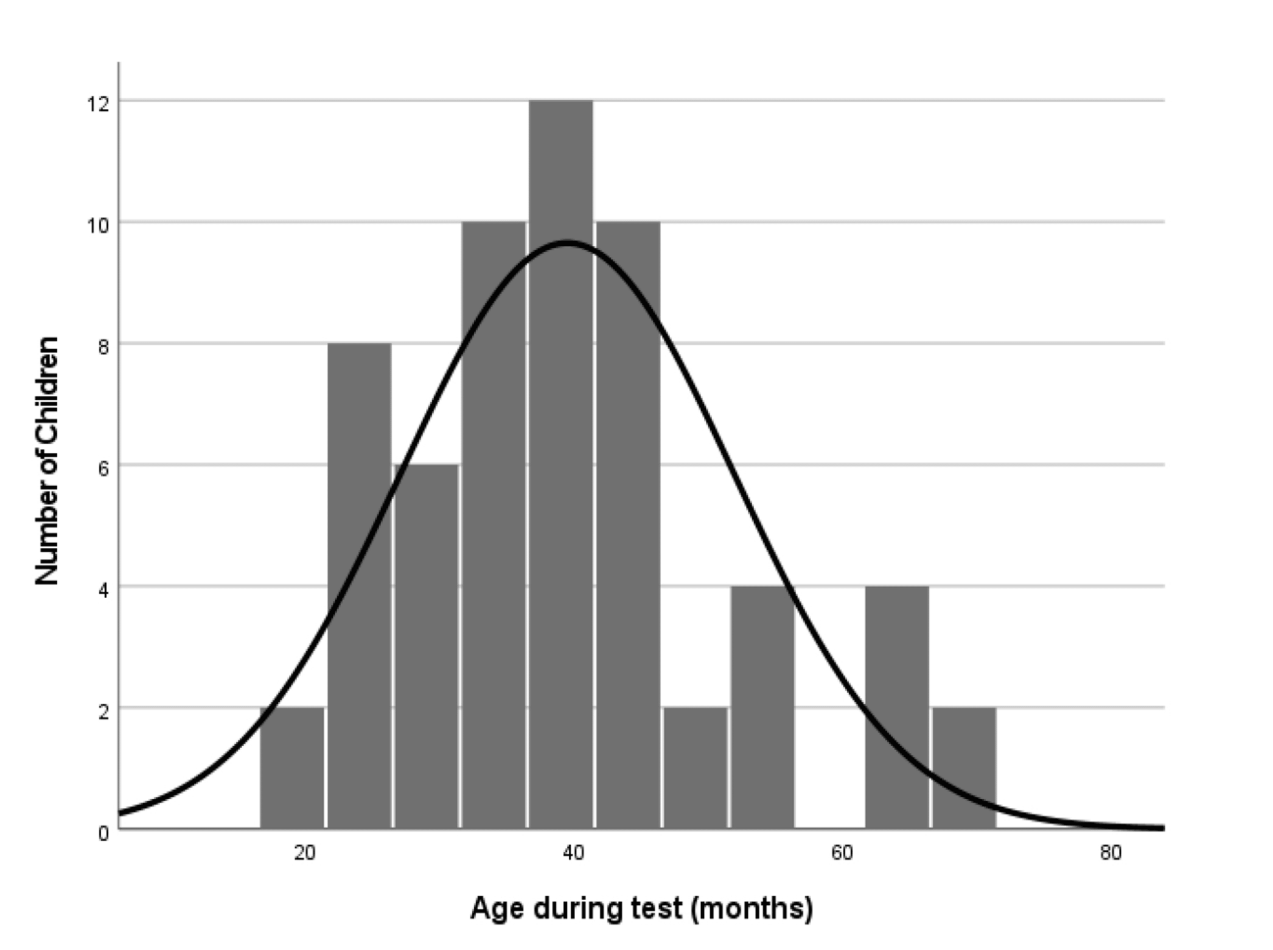
Age distribution of the 30 participants (60 ears) included in this study

The study sample comprised 30 children (21 males and nine females, totaling 60 ears) from the region of Western Greece. Of these, 20 children were diagnosed with ASD and speech delay, while the remaining 10 were non-ASD children with speech delay, serving as the control group. Participants’ ages (Figure 1) ranged from 19 to 68 months, with a median age of 38.5 months. Given the sample size, a total of 60 ears were tested. All children underwent examination using click ABR and chirp ASSR electrophysiological methods at the Audiology Unit of the Otorhinolaryngology Department at Karamandaneio Children’s Hospital in Patras between December 13, 2019 and December 6, 2023.

The non-ASD children were referred by pediatricians, otolaryngologists, and speech therapists, while the children with ASD were referred by pediatricians, otolaryngologists, speech therapists, child development specialists, child psychiatrists/psychologists, and child neurologists. Prior to the examination, all children had been assessed by one or more of these specialists, either in a public hospital or privately. The primary reason for the electrophysiological examination was the speech delay exhibited by the children, as well as their inability to undergo a behavioral examination due to their age and, in the case of the children diagnosed with ASD, behavioral challenges. Before the test was performed, parents were verbally briefed by the clinician regarding the procedure and the intended use of the results, after which they were asked to sign a written consent form. Table 1 summarizes the inclusion and exclusion criteria for the study.

**Table 1:** Inclusion and exclusion criteria for this research study ASD, autism spectrum disorder; OME, otitis media with effusion

Inclusion criteria	Exclusion criteria
Greek speaker	Bilingual speaker
Age range: 18-70 months	Other communication difficulties
Speech delay	Unofficial diagnosis of autism
Official diagnosis of ASD	Non-official diagnosis referral from a clinic
	Users of cochlear implants, hearing aids, etc.
	Co-occurring problems (e.g., dual diagnosis)
	Non-completion of the procedure due to technical reasons or impossibility/difficulty of the child falling asleep (e.g., a case where the child woke up before the completion of the examination)
	Congenital anomalies of the ear
	Children found having OME

On the day of the procedure, an otoscopic examination was conducted to assess the function of the outer and middle ear. If signs of ear infections (e.g., otitis) were observed, the examination was postponed, and the child was referred to an otolaryngologist for further evaluation. Additionally, a tympanometry test was performed prior to the examination: a 1,000 Hz tone for infants younger than seven months and a 226 Hz tone for infants older than seven months.

Typically developing children (control group with normal hearing) were initially diagnosed with speech delay before any audiological testing. The diagnosis of speech delay was based on an assessment of the child’s speech development, including the recognition of everyday objects, classification, response to various stimuli, object categorization, numerical skills, recognition and understanding of spatial and temporal concepts, imitation skills (e.g., fine and gross motor skills), spoken language description and expression, memory recall, visual-motor coordination, text comprehension, and more.

The diagnosis of speech delay and autism in children with ASD was made by certified clinicians using psychometric tools such as the Autism Diagnostic Observation Schedule-Generic (ADOS-G), the Autism Diagnostic Interview-Revised (ADI-R), and the Modified Checklist for Autism in Toddlers (M-CHAT), in addition to informal observations as mentioned earlier. It is important to note that the diagnosis of autism primarily relies on the observation of the individual, evaluation of developmental level, communicative interaction, socialization, functional and imaginative play, and behavior under various conditions. Finally, child psychiatrists and child development specialists use the DSM and ICD classification manuals for their diagnoses. Although the diagnosis of autism was not part of this study, it was a prerequisite that all children had received a formal diagnosis of ASD prior to the commencement of this research.

Sedation Procedure

Children were sedated with chloral hydrate, which was administered by a hospital nurse. At Karamandaneio Pediatric Hospital, the ABR and ASSR tests are conducted in children aged six months to five years under sedation with chloral hydrate (50 mg/ml). The initial dose, either orally or rectally, ranges from 50 to 70 mg/kg and is administered 30 minutes before the scheduled exam [15-17]. The medication is given by specialized nurses under the supervision of anesthesiologists in a safe room equipped with a medical monitor and resuscitation equipment. It typically took about 10-30 minutes for the children to become drowsy and fall asleep. During the examination, a hospital nurse continuously monitored the child’s oxygen levels, heart rate, and respiratory function. Upon completion of the test, the child was accompanied by their parents and placed under nurse supervision in a hospital room, where fluids and food were provided. Once the child was fully alert, they were permitted to return home with the clinic’s approval. The ABR test was performed first, followed by the ASSR test, with the total audiological examination lasting approximately 30-40 minutes. Depending on the child’s age, they were either lying on a bed or in a cot. The examination room was designed to promote sleep, with low noise levels, dim lighting, adequate heating, ventilation, air conditioning, proper electrical shielding, and minimal electrical interference.

Stimulation and recording parameters

ABR

The audiological examination began with the ABR test, and both ears of each child were tested. The procedure was conducted using the Eclipse EP25 System from Interacoustics (Middelfart, Denmark), with software version 3.03. After the child fell asleep, the skin was carefully cleaned using Nuprep® gel to reduce the electrode’s impedance, which can significantly affect the quality of the EEG [18].

Surface electrodes were then placed on the head, specifically one on the upper part of the forehead (Fz) for the non-inverting electrode, one on the lower part of the forehead (Fpz) as the ground electrode, and bilaterally on the mastoid (M1 for the left side and M2 for the right side) for the inverting electrodes. Sanibel snap disposable clip-on electrodes were used for both ABR and ASSR testing. The acceptable value for the absolute impedance of the electrodes was less than 5 kΩ, and the total interelectrode impedance remained within ± 2 kΩ throughout both tests.

A broadband click stimulus ranging from 200 Hz to 11 kHz with a duration of 100 μs was used during the examination. The stimulus was presented separately to each ear through EarTone ABR 50 Ω insert earphones with disposable foam tips, calibrated using an IEC 711 coupler, at a maximum intensity level of 80 dB nHL, delivered at a rate of 39.1 Hz per second with rarefaction polarity. The audiometer was calibrated according to the IEC 60645-1/ANSI S3.6 and IEC 60645-3 standards for auditory test signals [19]. The EEG signal was amplified via a preamplifier with filter settings of 100-3,000 Hz (6 dB/octave) and a gain of 80 dB, as instructed by the manufacturer. The time window was set to 15 ms.

Artifact rejection settings influence test efficiency, and the optimal level can vary based on test conditions [20]. For this study, artifact rejection was set to ± 40 μV with an automatically adjusted gain of 92 dB. The maximum number of sweeps used was 2,000, but the averaging process was automatically stopped once a distinct response was detected. Initially, the intensity was set to 70 dB nHL. If a clear response with all major waves (I, III, and V) at normal latency values was present, the intensity was reduced to 40 dB nHL. The test continued with intensity levels decreased by 10 dB until an approximate threshold was established, then adjusted by 5 dB up and down around the hearing threshold point. To ensure reliable results, the test was repeated at the first and last stimulus intensity levels, and averaging ceased once a repeatable response was identified. The lowest intensity level at which a repeatable wave V was detected was used to determine the hearing threshold, which was visually identified by the clinician based on the waveform peaks.

In cases where an ABR response could not be recorded at the upper intensity limits, two measurements were taken: one with positive polarity (condensation) and one with negative polarity (rarefaction) to detect cochlear microphonic (CM) and differentiate it from wave I [21]. Recordings showing only CM were excluded from the study. Additionally, a transient evoked OAE (TEOAE) test was conducted on all cases to rule out auditory neuropathy. For cases where CM was recorded, TEOAEs were also assessed. The TEOAE testing was performed using the Titan platform from Interacoustics, with software version 3.4.0 and settings as recommended by the manufacturer. The total recording time for the ABR test was approximately 10 minutes.

ASSR

For the ASSR testing, two channels were used, with the Eclipse EP25 ASSR System from Interacoustics, software version 1.2.3, serving as the recording device. All parameters were adjusted according to the specific software protocol for children who were asleep. The stimulus administered was the narrowband (octave-band) CE-Chirp (NB CE-Chirp), with carrier frequencies of 0.5, 1, 2, and 4 kHz, presented binaurally through EarTone ABR 50 Ω insert earphones with disposable foam tips. The CE-Chirp stimuli are described in detail by Elberling et al. [22]. The amplitude modulation for these frequencies was 100%, with modulation frequencies set as follows: 86, 90, 84, and 88 Hz for the right ear, and 94, 96, 92, and 95 Hz for the left ear, according to the Eclipse EP25 protocol. The narrowband CE-Chirp ASSR stimulus is particularly effective for accurately estimating audiometric thresholds, even at 0.5 kHz.

Testing began with an initial ASSR stimulus intensity of 60 dB nHL, except for children with moderate-to-severe hearing loss identified during the ABR testing, in which case the stimulus intensity was set 10 dB above the threshold determined by the ABR test. The maximum presentation level for all frequencies was 100 dB nHL.

The first stage of the ASSR test involved simultaneously presenting the four frequencies in each ear using the Multiple Auditory STEady-state Response (MASTER) technique, also known as the multiple frequency ASSR technique [23]. The ASSR thresholds for each frequency were established by adjusting the stimulus level after completing the initial stage. If the response approached the hearing threshold, the stimulus level was increased or decreased in 5 dB steps until the threshold was identified. The lowest level that met the significance criteria was used as the threshold. Additionally, the thresholds were determined using the correction factors from the Eclipse EP25 software, as indicated by previous research [24].

The chirp stimulus was delivered through the EarTone ABR 50 Ω insert earphones, which offer significant clinical advantages over traditional over-the-ear headphones. The direct coupling to the auditory canal enhances the performance and reliability of the audiometric tests. These insert earphones were calibrated to an IEC 711 coupler, and the signals were calibrated according to the IEC 60645-1/ANSI S3.6 standard for audiometers and the IEC 60645-3 standard for auditory test signals.

The EEG signal was amplified via a preamplifier with an 80 dB gain before being band-pass filtered between 33 and 6,000 Hz. The incoming signals were converted from analog to digital format using a 16-bit A/D converter with a 30 kHz sampling rate. Artifact rejection was set to ± 40 μV.

The Interacoustics software used for the ASSR testing does not have a set number of sweeps, as the system continues averaging until it detects a statistically significant response. Testing ends either when the maximum time limit (six minutes) is reached, yielding a “no response” decision, or when a significant response is detected. The software also allows for the selection of two levels of significance (p = 0.05 and p = 0.01), which correspond to “speed” and “accuracy” recording modes. For this study, the “accuracy” mode was used. The total time for the ASSR test was approximately 20-30 minutes.

This research was part of a doctoral thesis, and data collection was approved by the scientific council of Karamandaneio Children’s Hospital of Patras (14123, December 9, 2019) and the general assembly of the Department of Medicine, School of Health Sciences, University of Patras (1491/8658, March 27, 2019). Written consent was obtained from the parents of all participants.

Statistical analysis

Due to the lack of normal distribution in the data, nonparametric statistical analysis methods were employed, specifically Spearman’s rank correlation coefficient and the Mann-Whitney U test, to assess bivariate relationships among variables [25-27]. The deviation from normal distribution in the study’s data was indicated by the statistically significant results (p < 0.05) of the Shapiro-Wilk test for the metric variables, including the click ABR scores and chirp ASSR threshold average scores. Additionally, the sample size (n = 60 ears) and the absolute z-values of skewness and/or kurtosis for each metric variable exceeded the suggested threshold of 3.29, confirming the non-normality of the data distribution [28].

Statistical analyses were performed using IBM SPSS Statistics for Windows, Version 27.0 (Released 2020; IBM Corp., Armonk, NY, USA). In line with standard practice [29], a 5% significance level (p < 0.05) was applied. However, recognizing that statistical significance can be arbitrary and may not always reflect practical significance, particular attention was also given to effect sizes, such as the magnitude of correlation coefficients and the effect sizes for the U tests performed.

## Results

Table 2 presents the relationships between the click ABR and chirp ASSR threshold averages for the study sample. A statistically significant association was found between the click ABR and chirp ASSR threshold averages at 1,000, 2,000, and 4,000 Hz (rho = 0.316, p = 0.014), as well as at 2,000 and 4,000 Hz (rho = 0.277, p = 0.032). The strongest positive correlation, however, was observed between the two chirp ASSR threshold averages (rho = 0.971, p < 0.001).

**Table 2:** Nonparametric bivariate correlations (Spearman’s rho) between measures of hearing evaluation n = 60 ears Statistically significant correlations are indicated in bold. ABR, auditory brainstem response; ASSR, auditory steady-state response

		ABR	Average ASSR: 1 kHz, 2 kHz, and 4 kHz	Average ASSR: 2 kHz and 4 kHz
ABR	Rho	1		
	p-value	-		
Average ASSR: 1 kHz, 2 kHz, and 4 kHz	Rho	0.316	1	
	p-value	0.014	-	
Average ASSR: 2 kHz and 4 kHz	Rho	0.277	0.971	1
	p-value	0.032	0	-

The chirp ASSR threshold averages at 1,000, 2,000, and 4,000 Hz, as well as at 2,000 and 4,000 Hz, were compared to the click ABR thresholds. Since the click ABR typically corresponds to the best or average threshold within the 1,000-4,000 Hz range, these threshold averages were selected for comparison [30]. Given that the click stimulus operates within the high-frequency range, Swanepoel and Ebrahim [31] also found that the 1-4 kHz average exhibited the highest correlation, followed by the 2-4 kHz average.

Table 3 presents the impact of ASD on click ABR and chirp ASSR threshold averages. The results indicate that participants diagnosed with ASD show statistically significantly lower mean scores for both click ABR and chirp ASSR threshold averages compared to participants without ASD. The effect appears to be slightly more pronounced for the chirp ASSR threshold averages than for the click ABR.

**Table 3:** Mann-Whitney U tests of the effect of ASD on measures of hearing evaluation The effect size (r) is calculated using the formula r = Z / √n, which reflects the magnitude of each impact. ABR, auditory brainstem response; ASD, autism spectrum disorder; ASSR, auditory steady-state response

		N (ears)	Mean (dB)	SD	p-value	Effect size (r)
ABR	Speech delay and non-ASD	20	24	4.47	0.015	-0.32
	Speech delay and ASD	40	21.75	8.66		
Average ASSR: 1 kHz, 2 kHz, and 4 kHz	Speech delay and non-ASD	20	19.08	13.49	0.003	-0.39
	Speech delay and ASD	40	9.13	12.41		
Average ASSR: 2 kHz and 4 kHz	Speech delay and non-ASD	20	17.25	12.82	0.009	-0.34
	Speech delay and ASD	40	9.19	11.54		

## Discussion

The need for an efficient technique to calculate reliable hearing thresholds in difficult-to-test populations has long been a priority in pediatric audiology. Auditory-evoked potentials have been utilized in diagnostic audiology for over five decades, and considerable effort has been invested in the field of objective audiology to address this need. To date, the gold standard technique for determining hearing thresholds in infants has been the ABR procedure. Through broadband click and chirp stimuli, ABR can provide a general assessment of hearing thresholds in high-frequency ranges (2-4 kHz), while tone bursts offer specific frequency information.

On the other hand, ASSR has been used in audiology research centers for approximately four decades and has shown promise in addressing some of the limitations of ABR [32]. Research findings indicate that ASSR thresholds can predict pure-tone thresholds in infants and young children while they are asleep [33], which is consistent with our own results. Specifically, the ASSR test appears to have greater sensitivity in detecting hearing loss in participants with autism compared to ABR. Thus, ASSR should play an increasing role in the diagnostic evaluation and follow-up of infants who fail newborn hearing screenings. Notably, our results show that the correlation between frequency averages at 2 and 4 kHz, as well as at 1, 2, and 4 kHz, is not significantly different, with minimal fluctuations observed. Therefore, the choice of frequency pair seems inconsequential. When used alongside ABR, ASSR can provide additional information about the degree of any existing hearing loss [33]. Furthermore, ASSR holds promise for objectively adjusting hearing aids in individuals who cannot reliably respond to behavioral audiological tests.

In contrast to ASSR, which simultaneously evaluates four frequencies (500 Hz, 1,000 Hz, 2,000 Hz, and 4,000 Hz) using narrow-band chirp stimuli in both ears, ABR typically uses short-duration clicks, chirps, or tone bursts as stimuli [34]. In cases of mild to severe hearing loss, ABR is helpful for estimating hearing thresholds from 1,000 to 4,000 Hz. However, ASSR can also estimate hearing thresholds in individuals with severe to profound hearing loss, and in the 500 to 4,000 Hz range. During ABR recording, the examiner determines the presence or absence of a response by observing and evaluating the waveform process. As ABR approaches the hearing threshold, the decision regarding the presence or absence of a response becomes more challenging. In contrast, ASSR uses an objective, advanced statistical detection technique to determine hearing thresholds.

We should note that a comparison was made between the 40 ears of the ASD cases and the 20 ears of the controls. No comparison was made between the left and right ears, following the approach used in previous literature [30]. Two potential limitations of our study include the lack of a larger sample of children with ASD and the difficulty of performing behavioral audiological testing in these children due to diagnostic and behavioral challenges.

Since the implementation of the ASSR test in clinical audiological practice, efforts have been made to compare it with the already-established ABR test. However, to date, there are no studies in the international literature comparing these two electrophysiological methods in children with ASD, nor is there such research in Greece. This study aimed to compare the hearing thresholds obtained through these two objective audiological tests in children on the autism spectrum with varying degrees of hearing loss.

## Conclusions

Specific diagnostic tools for assessing hearing thresholds may significantly enhance the clinician’s understanding of ASD and demonstrate test-retest reliability. In this context, ABR and ASSR are methods that warrant further investigation. This study concludes that the ASSR test is a valid and reliable method for diagnosing hearing loss in children with ASD. The results also highlight the potential of ASSR as an adjunct to ABR testing. Equally significant is the finding that the hearing sensitivity of children on the autism spectrum is slightly better than that of children with normal hearing. These findings are crucial because the behavioral disorders characteristic of children with ASD often prevent reliable hearing evaluation using subjective methods. As such, ABR and ASSR offer substantial value in assessing hearing in children with ASD, both for clinical evaluations and research purposes.

The “gold standard” for diagnosing ASD remains a clinical diagnosis based on the clinician's best judgment. To ensure accuracy, this judgment should be informed by a combination of methods, including physical examination, hearing testing, child observation, parent interviews, and a comprehensive developmental history.
